# Variations in *ORAI1* Gene Associated with Kawasaki Disease

**DOI:** 10.1371/journal.pone.0145486

**Published:** 2016-01-20

**Authors:** Yoshihiro Onouchi, Ryuji Fukazawa, Kenichiro Yamamura, Hiroyuki Suzuki, Nobuyuki Kakimoto, Tomohiro Suenaga, Takashi Takeuchi, Hiromichi Hamada, Takafumi Honda, Kumi Yasukawa, Masaru Terai, Ryota Ebata, Kouji Higashi, Tsutomu Saji, Yasushi Kemmotsu, Shinichi Takatsuki, Kazunobu Ouchi, Fumio Kishi, Tetsushi Yoshikawa, Toshiro Nagai, Kunihiro Hamamoto, Yoshitake Sato, Akihito Honda, Hironobu Kobayashi, Junichi Sato, Shoichi Shibuta, Masakazu Miyawaki, Ko Oishi, Hironobu Yamaga, Noriyuki Aoyagi, Megumi Yoshiyama, Ritsuko Miyashita, Yuji Murata, Akihiro Fujino, Kouichi Ozaki, Tomisaku Kawasaki, Jun Abe, Mitsuru Seki, Tohru Kobayashi, Hirokazu Arakawa, Shunichi Ogawa, Toshiro Hara, Akira Hata, Toshihiro Tanaka

**Affiliations:** 1 Laboratory for Cardiovascular Diseases, Center for Integrative Medical Sciences, RIKEN, Yokohama, Japan; 2 Department of Pediatrics, Nippon Medical School, Tokyo, Japan; 3 Department of Pediatrics, Graduate School of Medical Sciences, Kyushu University, Fukuoka, Japan; 4 Department of Pediatrics, Wakayama Medical University, Wakayama, Japan; 5 Department of Pediatrics, Tokyo Women’s Medical University Yachiyo Medical Center, Yachiyo, Japan; 6 Department of Pediatrics, Graduate School of Medicine, Chiba University, Chiba, Japan; 7 Department of Cardiology, Chiba Children’s Hospital, Chiba, Japan; 8 Department of Pediatrics, Toho University Medical Center, Omori Hospital, Tokyo, Japan; 9 Department of Pediatrics, Kawasaki Medical School, Kurashiki, Japan; 10 Department of Molecular Genetics, Kawasaki Medical School, Kurashiki, Japan; 11 Department of Pediatrics, Fujita Health University, Toyoake, Japan; 12 Department of Pediatrics, Dokkyo Medical University Koshigaya Hospital, Koshigaya, Japan; 13 Department of Occupational Therapy, International University of Health and Welfare, Okawa, Japan; 14 Department of Pediatrics, Fuji Heavy Industry Health Insurance Society Ota Memorial Hospital, Ota, Japan; 15 Department of Pediatrics, Asahi General Hospital, Asahi, Japan; 16 Department of Pediatrics, Funabashi Municipal Medical Center, Funabashi, Japan; 17 Department of Pediatrics, Kinan Hospital, Tanabe, Japan; 18 Department of Pediatrics, Hashimoto Municipal Hospital, Hashimoto, Japan; 19 Department of Pediatrics, Naga Hospital, Kinokawa, Japan; 20 Department of Pediatrics, Wakayama Rosai Hospital, Wakayama, Japan; 21 Department of Pediatrics, Hidaka General Hospital, Gobo, Japan; 22 Department of Pediatrics, Izumiotsu Municipal Hospital, Izumiotsu, Japan; 23 Department of Pediatrics, Sendai City Hospital, Sendai, Japan; 24 Department of Pediatric Surgery, Keio University School of Medicine, Tokyo, Japan; 25 Japan Kawasaki Disease Research Center, Tokyo, Japan; 26 Department of Allergy & Immunology, National Center for Child Health and Development, Tokyo, Japan; 27 Department of Pediatrics, Gunma University School of Medicine, Maebashi, Japan; 28 Department of Development Strategy, Center for Clinical Research and Development, National Center for Child Health and Development, Tokyo, Japan; 29 Department of Public Health, Graduate School of Medicine, Chiba University, Chiba, Japan; 30 Department of Human Genetics and Disease Diversity, Tokyo Medical and Dental University, Tokyo, Japan; University of Hull, UNITED KINGDOM

## Abstract

Kawasaki disease (KD; MIM#61175) is a systemic vasculitis syndrome with unknown etiology which predominantly affects infants and children. Recent findings of susceptibility genes for KD suggest possible involvement of the Ca^2+^/NFAT pathway in the pathogenesis of KD. ORAI1 is a Ca^2+^ release activated Ca^2+^ (CRAC) channel mediating store-operated Ca^2+^ entry (SOCE) on the plasma membrane. The gene for ORAI1 is located in chromosome 12q24 where a positive linkage signal was observed in our previous affected sib-pair study of KD. A common non-synonymous single nucleotide polymorphism located within exon 2 of *ORAI1* (rs3741596) was significantly associated with KD (*P* = 0.028 in the discovery sample set (729 KD cases and 1,315 controls), *P* = 0.0056 in the replication sample set (1,813 KD cases vs. 1,097 controls) and *P* = 0.00041 in a meta-analysis by the Mantel-Haenszel method). Interestingly, frequency of the risk allele of rs3741596 is more than 20 times higher in Japanese compared to Europeans. We also found a rare 6 base-pair in-frame insertion variant associated with KD (rs141919534; 2,544 KD cases vs. 2,414 controls, *P* = 0.012). These data indicate that *ORAI1* gene variations are associated with KD and may suggest the potential importance of the Ca^2+^/NFAT pathway in the pathogenesis of this disorder.

## Introduction

Kawasaki disease (KD; MIM #611775) is an acute febrile illness which predominantly affects infants and children younger than 5 years of age [1;2]. Principal symptoms of KD are high fever, bilateral conjunctival congestion, changes in the appearance of the lips and oral cavity, skin rash, erythema and indurative edema of hands and feet, and cervical lymphadenopathy. Although KD is a self-limited disorder, cardiac complication represented by coronary artery aneurysms occurs in 20–25% of the patients if untreated [3]. Intravenous immunoglobulin (IVIG) therapy has proven to be effective in preventing coronary artery lesions (CALs) [4]; however, 10–15% of patients poorly respond to the treatment and are at high risk for developing CALs. Currently, KD is a leading cause of acquired heart diseases in children in developed countries.

Based on observations of its seasonality in incidence and previous epidemics experienced in Japan, it is believed that infectious agents may play an important role in the pathogenesis of the disease. However, after more than 40 years since Kawasaki first described the disease [1], the etiology still remains unknown. Meanwhile, a higher prevalence in children of Asian ancestry [5;6] and evidence of familial aggregation of the disease [7;8] have strongly indicated an involvement of genetic susceptibility. Thus, the identification of genetic factors contributing to the inter-ethnic and inter-individual difference in susceptibility to KD will help to clarify disease etiology.

A genome-wide linkage analysis by the affected sib-pair method in KD previously identified 10 chromosomal regions with nominal evidence of linkage [9]. In subsequent association studies using single nucleotide polymorphisms (SNPs), two susceptibility loci for KD were successfully identified [10;11]. One is *ITPKC* on 19q13.2 encoding inositol 1,4,5-trisphosphate 3-kinase C which catalyzes the phosphorylation of inositol 1,4,5-trisphosphate (IP3) leading to the down regulation of signal transduction along the Ca^2+^/NFAT pathway. The second locus is *CASP3* on 4q35 which encodes CASPASE3, a key molecule involved in apoptosis of immune cells. CASPASE3 was also reported to cleave nuclear factor of activated T-cells (NFAT) c2 [12] and the receptor for IP3 (ITPR1) [13], major components in the Ca^2+^/NFAT pathway signal transduction, as its substrates in T-cells. In this study, we focused on *ORAI1*, a CRAC channel that plays a key role in the SOCE mechanism on which various immune cells rely for activation of the Ca^2+^/NFAT pathway. *ORAI1* is a positional candidate gene of KD located at the 12q24 region where the highest linkage signal (MLS = 2.69) was observed in the previous linkage study [9].

## Materials and Methods

### Ethics statement

The ethical committees or institutional review boards at RIKEN (RIKEN Yokohama Campus Ethics Committee), Chiba University (Biomedical Research Ethics Committee of the Graduate School of Medicine, Chiba University), Nippon Medical School (Nippon Medical School Ethics Committee for Human Genome / Gene Analysis Research), Kyushu University (Kyushu University Institutional Review Board for Human Genome / Gene Research), Wakayama Medical University (Research Ethics Committee of Wakayama Medical University), Tokyo Women’s Medical University (Tokyo Women's Medical University Genome Ethics Committee), Chiba Children’s hospital (Institutional Review Board of Chiba Children's Hospital), Toho University (Human Research Ethics Committee of Toho University Faculty of Medicine), Kawasaki Medical School (Research Ethics Committee of Kawasaki Medical School and Hospital), Fujita Health University (the Ethical Review Boards for Human Genome Studies at Fujita Health University), Dokkyo Medical University (Bioethical Committee of Dokkyo Medical University), Fuji Heavy Industry Health Insurance Society Ota Memorial Hospital (Fuji Heavy Industry Health Insurance Society Ota Memorial Hospital Ethics Committee), Asahi General Hospital (Ethics Committee of Asahi General Hospital), Funabashi Municipal Medical Center (Funabashi Municipal Medical Center Ethics Committee), Kinan Hospital (Ethics Committee of Kinan Hospital), Naga Hospital (Ethics Committee of Naga Hospital), Wakayama Rosai Hospital (Wakayama Rosai Hospital Ethics Committee), Hidaka General Hospital (Ethics Committee of Hidaka General Hospital), Izumiotsu Municipal Hospital (Izumiotsu Municipal Hospital Ethics Committee), Sendai City Hospital (Sendai City Hospital Ethics Committee), Keio University School of Medicine (Keio University School of Medicine, An Ethical Committee), National Center for Child Health and Development (the Ethics Committee of National Center for Child Health and Development), Gunma University (Genome Ethics Committee at Gunma University Graduate School of Medicine) and Hashimoto Municipal Hospital (Ethics Committee of Hashimoto Municipal Hospital) approved the study. We obtained written informed consent from all the participants. As KD is a childhood disease and patients were infants and children at enrollment, in most cases written informed consent was obtained from the patients’ parents. When the patients were aged 16 to 20 years, we obtained written informed consent from both the patients themselves and their parents.

### Samples

We recruited 2,544 KD patients from several medical institutions in Japan. The control subjects of Japanese healthy adults were obtained from the Osaka-Midosuji Rotary Club, Osaka (n = 940), the Health Science Research Resources Bank, Osaka (n = 950) and from Keio University (n = 374). Patients with disorders unrelated to KD (n = 168) from Nippon Medical School were also enrolled as control subjects.

### Selection of *ORAI1* as a positional candidate gene to be studied

**Fig 1:**
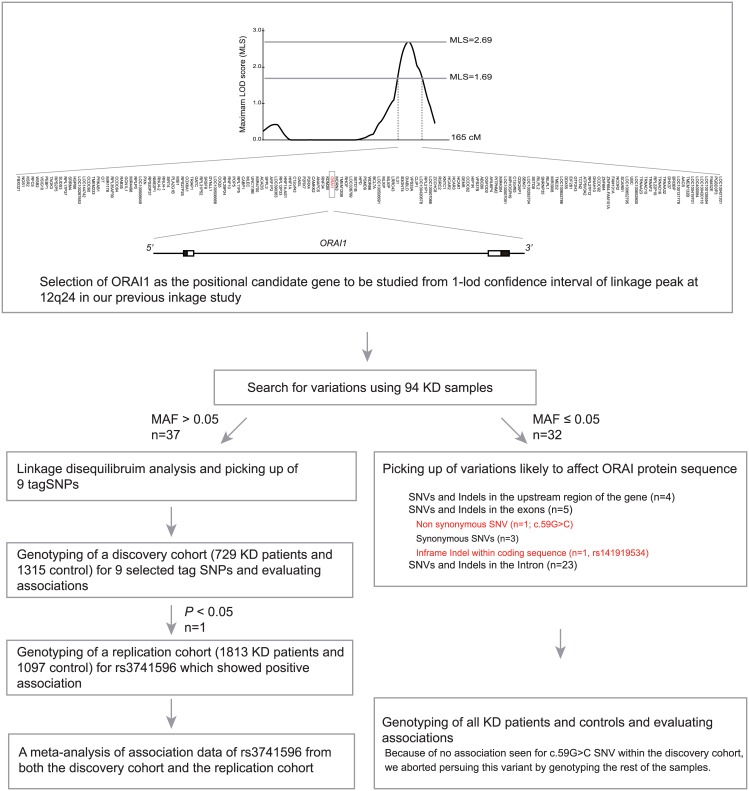
Flow of the study.

Based on the updated gene mapping information, we newly considered genes located within 1-lod confidence interval in linkage position on chromosome12 identified through our previous work (NC_000012.11: from 117.5 Mb– 127 Mb; Fig 1) [9]. Among the 151 genes fulfilling this criteria, we selected the *ORAI1* gene, which is located near the center of the linkage area (122.1Mb), as a positional candidate gene for this study.

### Re-sequencing and genotyping

We re-sequenced the *ORAI1* genomic region (NC_000012.11: from nt 122,062,619 to 122,076,990) using 94 KD subjects. To identify variants efficiently, the sample panel for re-sequencing consisted of mostly probands of familial KD cases recruited in our previous affected sib-pair study [9]. The number of samples was determined to enable detection of variants with minor allele frequencies as low as 0.01. Linkage disequilibrium among 37 variants with minor allele frequency greater than 0.05 was evaluated by Haploview 4.2 software. Selection of tag SNPs were performed by using the tagger option of the software with an r-squared threshold of 0.80. Genotyping of KD cases and controls for the SNPs were carried out by using the Invader assay as described previously [14]. Insertion / deletion variants were genotyped by direct sequencing.

### Statistical analysis

Association of the tag SNPs and KD was evaluated using the Pearson’s chi-square test. A meta-analysis of association data for rs3741596 in both the discovery cohort and replication cohort was conducted using the Mantel-Haenszel method. Fisher’s exact test was employed to assess association of the rare genetic variants (rs141919534 and c.59G>C) and KD. Conditional logistic regression analysis was conducted to see whether observed association of the SNPs represented by rs3741596 could be explained by linkage disequilibrium with rs76753792, the most significant SNP in the group. The Pearson’s chi-square test and meta-analysis with Mantel-Haenszel method were conducted using Microsoft Excel 2010 software. Fisher’s exact test and logistic regression analysis were conducted using the R version 2.15.2 statistical environment.

### *In silico* prediction of the functional effects of the variants

We used the Variant Effect Predictor web tool [15] to evaluate the impact of amino acid changes of ORAI1 on its protein function. miRNA target sequences within the 3’-UTR of *ORAI1* mRNA and the impact of the nucleotide changes within the targets was predicted using the mrSNP web service [16].

## Results

**Fig 2:**
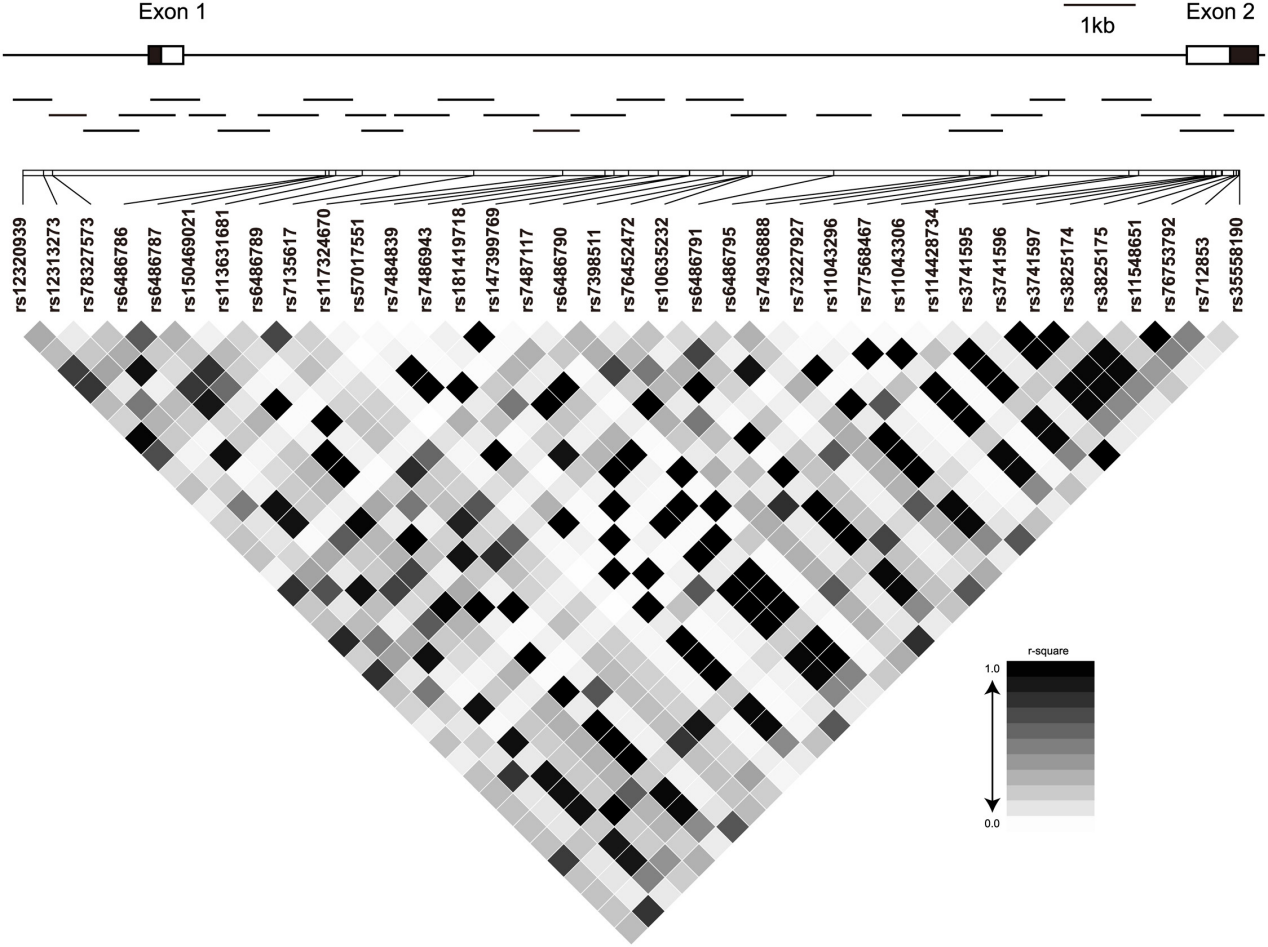
Linkage disequilibrium map of the common variants around the *ORAI1* gene. Upper: Genomic structure of the *ORAI1* gene. Middle: Positions and sizes of PCR amplicons. Lower: Results of pairwise LD analyses of the identified variants with minor allele frequencies greater than 0.05 (lower). r-squared values for each variant pair are presented in grayscale.

The experimental flow of this study was shown in Fig 1. Re-sequencing of the *ORAI* genomic region resulted in the identification of 69 variants (S1 Table). A linkage disequilibrium analysis including 37 polymorphisms with minor allele frequencies larger than 0.05 revealed 9 groups of polymorphisms which showed strong linkage disequilibrium (r^2^ > 0.8; Fig 2). To evaluate the association of these common polymorphisms with KD efficiently, we selected one representative SNP from each group and genotyped 730 KD cases and 1,318 controls at these loci. In this screening, one tag SNP (rs3741596) representing a group with 10 SNPs showed a nominal association (OR = 1.19, 95%CI 1.02–1.40, *P* = 0.028; Table 1). We then examined the association of rs3741596 in another case-control series (1,813 KD cases and 1,097 controls) for validation. As shown in Table 2, rs3741596 showed a similar association (OR = 1.22, 95%CI 1.06–1.40, *P* = 0.0056) and a meta-analysis of data from both discovery and validation cohorts indicated a statistically significant combined result (OR = 1.21, 95%CI 1.09–1.34 *P* = 0.00041). Analysis of the 9 other SNPs tagged by rs3741596 among the initial screening case-control series showed the same trend of association for all variants and only minor differences in odds ratios and *P* values (S2 Table). The lowest *P* value was observed for rs76753792 located within the 3’ untranslated region (UTR) of the gene, and the nucleotide change was predicted to alter binding affinity of miRNAs to the surrounding mRNA sequence (S3 Table). However, conditional logistic regression analyses did not indicate this SNP to be superior over the other 9 SNPs (data not shown).

**Table 1:** Association of tagging SNPs in the *ORAI1* region with KD.

dbSNP ID	Chromosomal location ^a^	position on NT_022792.17	Alleles		Samples	Genotype distribution						Statistical analysis ^f^			Position in gene
			1	2		11	12	22	Total	MAF ^b^	HWEP ^c^	OR ^d^	95% C.I. ^e^	P	
rs12313273	122063010	12639540	C	T	KD	43	291	394	728	0.26	0.24	1.02	0.88–1.18	0.801	5' flanking (-1465base)
					Control	75	500	698	1273	0.26					
rs6486789	122067972	12644502	T	C	KD	160	369	198	727	0.47	0.54	1.11	0.97–1.26	0.129	intron1 (IVS1+3016)
					Control	312	649	315	1276	0.50					
rs117324670	122070246	12646776	A	T	KD	4	112	613	729	0.082	0.97	1.03	0.82–1.30	0.797	intron1 (IVS1+5290)
					Control	9	197	1064	1270	0.085					
rs7484839	122070961	12647491	T	C	KD	0	109	614	723	0.075	0.0057	1.05	0.82–1.34	0.724	intron1 (IVS1+6005)
					Control	0	182	1076	1258	0.072					
rs7486943	122071163	12647693	T	C	KD	520	193	14	727	0.15	0.73	1.00	0.84–1.20	0.984	intron1 (IVS1+6207)
					Control	919	325	31	1275	0.15					
rs3741595	122079189	12655719	T	C	KD	32	266	432	730	0.23	0.81	1.02	0.88–1.19	0.763	exon2 (c.546C>T; p.I182I)
					Control	66	449	794	1309	0.22					
rs3741596	122079295	12655825	A	G	KD	444	252	33	729	0.22	0.62	1.19	1.02–1.40	0.028	exon2 (c.652A>G; p.S218G)
					Control	867	398	50	1315	0.19					
rs3825175	122079441	12655971	T	C	KD	101	334	290	725	0.37	0.72	1.08	0.95–1.23	0.256	exon2 (c.798T>C; p.T266T)
					Control	195	632	491	1318	0.39					
rs712853	122079668	12656198	A	G	KD	255	363	108	726	0.40	0.94	1.03	0.90–1.18	0.658	3' UTR ^g^
					Control	469	606	194	1269	0.39					

^a^ Chromosomal locations were based on GRCh37 Patch Release 13 (GRCh37. p13).

^b^ Minor allele frequency.

^c^ P values for Hardy-Weinberg equilibrium in controls.

^d^ Odds ratio.

^e^ Confidence interval.

^f^ Associations in allelic model were evaluated by using chi-square test.

^g^ Untranslated region.

**Table 2:** Follow-up association study for rs3741596.

	Replication case—control series ^a^								Combined ^b^		
	AA	AG	GG	Total	MAF ^c^	OR ^d^	95% C.I. ^e^	*P*	OR	95% C.I.	*P*
KD	1172	577	64	1813	0.19	1.22	1.06–1.40	0.0056	1.21	1.09–1.34	0.00041
Control	760	311	26	1097	0.17						

^a^ Association in allelic model was evaluated by using chi-square test.

^b^ A meta-analysis of data from both discovery and validation cohorts was conducted with Mantel-Haenszel method.

^c^ Minor allele frequency.

^d^ Odds ratio.

^e^ Confidence interval.

Next, we investigated the possible involvement of rare genetic variants of this gene in KD susceptibility. We identified 32 variants with minor allele frequencies of less than 0.05 including 4 in the upstream region of the gene, 5 in exons and 23 in introns (S1 Table). There were no exonic and intronic variants within known consensus sequences of splicing acceptor or donor sites. Among the 5 exonic variants, one single nucleotide variant (SNV) (c.59G>C; p.G20A) and one 6-bp insertion variant (rs141919534, c.126-7insCCGCCA; p.42A_p.43PinsPP) appeared to alter ORAI1 protein sequence, and the other 3 included a synonymous SNV and 2 3’-UTR variants. We further investigated the c.59G>C SNV and rs141919534 with respect to directly altering the ORAI protein sequence. We first assessed the association of rs141919534 because the 6-bp insertion results in elongation of a proline repeat located within the N-terminal cytoplasmic domain of ORAI1 (S1 Fig) which is thought to directly interact with STIM1, the endoplasmic counterpart of ORAI1 [17;18]. We genotyped all cases and controls for this variation and found that the 6-bp insertion was over-represented among KD patients (OR = 3.80, 95%CI 1.23–15.64, *P* = 0.012; Table 3). Results of haplotype inference indicated that the 6-bp insertion had occurred on a chromosome bearing the major allele of rs3741596 (S2 Fig). No haplotype effect was observed between these two variants when examining haplotype associations (data not shown). In contrast, we failed to detect an association for the c.59G>C SNV, also located within N terminal cytoplasmic domain, in the case and control panel used in the initial screening of the tag SNPs (S4 Table).

**Table 3:** Association of rs141919534 with KD.

	WT ^a^ / WT	WT/insCCACCG	insCCACCG/insCCACCG	Total	MAF ^b^	OR ^c^	95% C.I. ^d^	*P*
KD	2528	16	0	2544	0.0031	3.80	1.23–15.64	0.012
Control	2410	4	0	2414	0.00083			

Association in allelic model was evaluated by using Fisher's exact test.

^a^ Wild type.

^b^ Minor allele frequency.

^c^ Odds ratio.

^d^ Confidence interval.

## Discussion

ORAI1 was identified as a membrane bound Ca^2+^ channel protein essential for SOCE of T lymphocytes [19]. ORAI1 is activated by direct interaction with STIM1 which is expressed on the endoplasmic reticulum (ER) membrane and acts as a sensor of Ca^2+^ store depletion in the ER. When inositol 1,4,5-trisphosphate (IP_3_) generated by hydrolysis of phosphatidylinositol 4,5-bisphosphate (PIP_2_) in response to signals from stimulated cell surface receptors (T-cell, B-cell, Immunoglobulin G Fc receptors, etc.) binds to the IP_3_ receptor (IP3R) on the ER membrane, changes in conformation of the IP3R molecule and tertiary structure of their tetramer complex are induced, and flux of Ca^2+^ stored within the ER lumen through IP3R is evoked. When depletion of stored Ca^2+^ in the ER is sensed by STIM1 with its EF-hand domain, multiple STIM1 and ORAI1 molecules interact directly and form complexes. This complex formation leads to conformational changes of the pore-lining transmembrane domain of ORAI1 to gate the channel for Ca^2+^ entry from the extra cellular space into the cytoplasm. Increase of cytosolic Ca^2+^ leads to the activation of calcineurin and the dephosphorylation and nuclear translocation of NFAT. This series of reactions, referred to as SOCE [20–23], is essential for T-cell activation, and deficiency of either ORAI1 or STIM1 causes autosomal recessive primary immune deficiency syndromes (MIM: 612782 or 612783) [24;25]. Analysis of mice homozygously expressing nonfunctional Orai1 in the hematopoietic tissue revealed a critical role for Orai1 in T-cell mediated autoimmunity and allograft rejection [26]. It is known that ORAI1 and STIM1 are expressed also in other hematopoietic cells such as B-cells [27], dendritic cells [28], neutrophils [29], NK cells [30], platelets [31] and mast cells [32;33]. ORAI1 and STIM1 regulate proliferation, apoptosis and metastasis of various cancer cells [34]. Involvement of these two molecules in activation and proliferation of human umbilical vein endothelial cells has also been reported [35;36]. Thus, SOCE is not recognized as a phenomena restricted to the immune system. Given that many types of immune and vascular endothelial cells are activated and contribute to KD pathogenesis, dysregulation of the mechanism could be one pathophysiological basis of KD vasculitis.

The previous identification of KD-associated SNPs within *ITPKC* and *CASP3* and their presumed role as negative regulators of the Ca^2+^/NFAT pathway led us to suspect genes involved in this pathway as candidate susceptibility loci for KD. *ORAI1*, which plays a pivotal role in SOCE, a form of Ca^2+^/NFAT signal transduction in the abovementioned types of cells, is located near the highest linkage peak (12q24) observed in our previous affected sib-pair study [9]. In the current study, we observed a KD association with one common variant (rs3741596) and a rare polymorphism (rs141919534) of *ORAI1* in the Japanese population.

SNPs of the *ORAI1* gene region and/ or their combinations have been associated with other inflammatory diseases as well, such as atopic dermatitis, ankylosing spondylitis or rheumatoid arthritis and calcium nephrolithiasis [37–40]. A previous study described an association of the rs3741596 G allele with susceptibility to atopic dermatitis in Japanese [34]. Interestingly, in the Taiwanese population, it was reported that rs3741596 G allele is much less frequent (< 1%) than in Japanese (17%—19% in this study), and tag SNPs of *ORAI1* were not associated with KD [41]. Considering the wide variety of tissues or cells which express *ORAI1* mRNA under different regulatory mechanisms and differences in cell types playing major roles in disease pathogenesis, it might be possible that responsible variants differ from disease to disease and, conversely, the Taiwanese population has no common *ORAI1* variants relevant to KD. rs3741596 alters translation of the 218th amino acid within the 2nd extra cellular loop of ORAI1 from serine to glycine (S1 Fig). In contrast to the known importance of the first extracellular loop in Ca^2+^ selectivity [42;43], the precise role of the 2nd extracellular loop is not known. Less conservation of the amino acid sequence around rs3741596 across species and the result of *in silico* prediction of the impact of the sequence alteration on protein function (S3 Fig) do not support rs3741596 as the casual variant. An LD analysis of the genotype data from the 1000 Genomes database revealed that there are 82 variants tightly linked to rs3741596 (r^2^ > 0.8) in the Japanese population which are distributed across a 100-kb genomic region (S5 Table). Within this 100-kb region, there is another gene whose expression or function could be affected by the associated variant(s). Function of the gene product, membrane occupation and recognition nexus repeat containing 3 (MORN3), has not been functionally characterized. However in mice, from its specific expression in testis and its property of binding to meiosis expressed gene 1 (Meg1), a regulator of spermatogenesis, it has been suggested that Morn3 also plays a role in sperm formation [44]. Based on this suspected biology, currently there is little evidence to consider *MORN3* as a candidate gene in KD susceptibility.

The 6-bp insertion allele of rs141919534 is exclusively linked to the A allele at rs3741596, the non-risk allele at this locus (S2 Fig), indicating that the observed association of this insertion / deletion variant is not a spurious one due to LD between these two sites. No other variant in strong LD (r^2^ > 0.8) with rs141919534 was identified in the analysis of 1000 Genomes data. Taken together, although further details remain to be elucidated, we concluded that *ORAI1* is a novel susceptibility gene for KD. At present, the precise impact of the 2 amino acid elongation is not clear. In light of the possible importance of the N terminal cytoplasmic domain of ORAI1 in interaction with STIM1 [17;18] and marked activation of various immune cells expressing ORAI1 in the acute phase of KD [45], it is likely that tertiary structure modified by the 2 amino acid elongation results in up-regulation of the affinity between STIM1 and ORAI1, which in turn makes the cells prone to activation. Involvement of SOCE in regulating *Cyclooxygenase-2* (*COX-2*) gene expression in colorectal cancer cells has been documented [46;47]. Notably, cyclooxygenases is targeted by Aspirin, a non-steroidal anti-inflammatory agent administered to most of the KD patients as a part of standard treatment. It is also possible that the *ORAI1* variants play a significant role in mechanisms other than SOCEs. In neutrophils, which are markedly activated in the acute phase of KD and whose infiltration into the vascular wall has been considered as a major cause of vascular damage in early stages [48], STIM1 mediates SOCE following tyrosine kinase or G protein coupled receptor signaling [49]. However, it is also known that ORAI1 mediates C5a induced neutrophil migration independently from STIM1 and SOCE [50]. Further investigation is warranted to evaluate the impact of the variant on ORAI1 function as well as on disease pathogenesis.

Epidemiological findings have indicated that notable predilection of KD to East Asian ethnicities attribute to genetic background rather than geographic factors. Marked differences in KD risk by genetic background can be explained by differences in allele frequencies of susceptibility loci. We believe *ORAI1* is likely one genetic factor accounting for the observed inter-ethnic difference between children of Japanese and European ancestry. Based on 1000 Genomes data, the 83 variants associated with this locus were rare in the CEU populations when compared to the JPT populations in which minor alleles were nearly 20 times more frequent (S5 Table). Interestingly, as reported in the 1000 Genomes, the risk allele of rs3741596 (G) is considered to be an ancestral allele of this SNP, but is almost absent in gene pools in populations other than East Asians and those of African descent (S4 Fig). It is not clear whether the skewed allele distribution is due to difference in selection pressure among areas or to some other event such as a population bottleneck. However, it is highly probable that the rs3741596 G allele originated from a founder haplotype because the LD pattern between rs3741596 and other variants are conserved (S5 Table).

None of the 83 variants were part of the genotyping microarrays used in previous GWAS studies of KD. The insufficient coverage of the genomic region containing *ORAI1* by the SNP arrays is likely why associations at this locus had not been detected in our previous GWAS [51]. Insufficient statistical power in previous GWAS due to limits in sample sizes (several hundreds) have contributed to missed associations. However, it is also possible that there are a number of susceptibility genes for KD which have not been identified for the same reason as *ORAI1*. To our knowledge, *ORAI1* is the first gene of which both common and rare variants confer susceptibility to KD. Recently, increased attention has been given to rare genetic variants as a source of missing heritability. A genome-wide rare variant association study seems, at least at this moment, unrealistic considering the estimated number of samples required for a well-powered study (> 25,000) [52] and its enormous cost. Thus, investigating known susceptibility genes, as well as genes that directly or indirectly interact with them, may be an effective way of identifying rare variants related to KD.

In conclusion, we identified common and rare variants of *ORAI1* genes associated with KD. Further investigation of the role of the gene in the pathophysiology of KD is warranted.

## Supporting Information

S1 Fig A diagram of ORAI1 four trans-membrane protein and the positions of the three variants affecting ORAI1 protein sequence. (PDF)

S2 Fig Haplotypes and genotype combinations with two associated variants of the *ORAI1* gene in this study. (PDF)

S3 Fig Prediction of impact of the amino acid sequence alterations on ORAI1 function. (PDF)

S4 Fig Distribution of rs3741596 alleles in HapMap populations. (PDF)

S1 Table List of the identified variants. (XLS)

S2 Table Association results of the SNPs tagged by rs3741596 with KD. (XLS)

S3 Table miRNAs which bind differently to the surrounding sequences of the 3'-UTR SNPs tagged with rs3741596 (XLS)

S4 Table Association result of the c.59G>C variant of *ORAI1* gene with KD. (XLS)

S5 Table Frequencies and positions of the group of variants tagged by rs3741596. (XLS)
