# Supplementary material for: Variations in ORAI1 Gene Associated with Kawasaki Disease
Source: PLoS One. 2016 Jan 20;11(1):e0145486. doi: 10.1371/journal.pone.0145486 (PMC4720480; doi:10.1371/journal.pone.0145486)
Supplement: S1 Fig — (PDF) [file pone.0145486.s001.pdf]

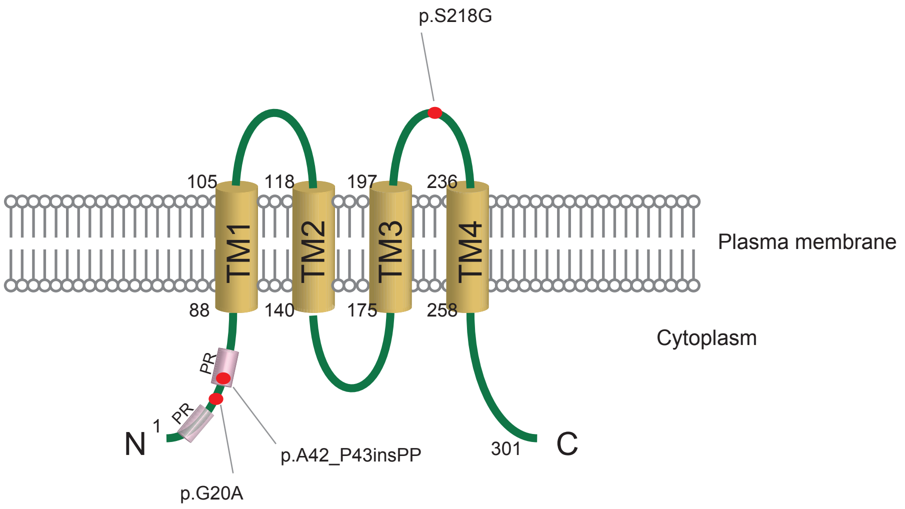

S1 Fig. A diagram of ORAI1 four trans-membrane protein and the positions of the three variants affecting ORAI1 protein sequence.

A schema of ORAI1 protein and the positions of the two proline rich regions (PR) in the N-terminal cytoplasmic region are shown. Numbers represents aminoacid positionss for both starts and ends of ORAI1 protein sequence as well as of four transmembrane regions (TM). Red dots are indicating positions of the aminoacids which are altered by the non-synonymous SNVs or the inframe insertion.
