# Supplementary material for: Variations in ORAI1 Gene Associated with Kawasaki Disease
Source: PLoS One. 2016 Jan 20;11(1):e0145486. doi: 10.1371/journal.pone.0145486 (PMC4720480; doi:10.1371/journal.pone.0145486)
Supplement: S2 Fig — (PDF) [file pone.0145486.s002.pdf]

a

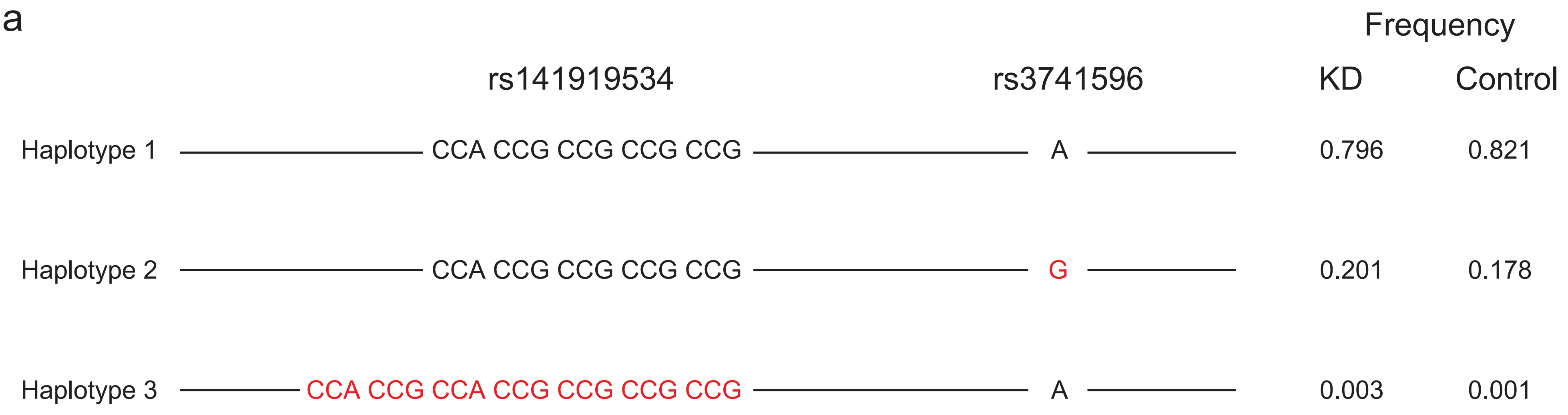

b

KD		rs141919534		
		WT/WT	WT/ins6b	ins6b/ins6b
rs3741596	AA	0.630	0.0060	0
	AG	0.325	0.00040	0
	GG	0.038	0	0

Control		rs141919534		
		WT/WT	WT/ins6b	ins6b/ins6b
rs3741596	AA	0.674	0.0013	0
	AG	0.293	0.00042	0
	GG	0.032	0	0

S2 Fig. Haplotypes and genotype combinations with two associated variants of the *ORAI1* gene in this study.
a. Haplotypes comprised with rs141919534 and rs3741596 and their frequencies in both KD cases and controls.
Risk alleles at both variants are in rubricated. **b.** Frequencies of Genotype combinations. WT: wild type, ins6b: insertion of CCACCG .
