# Supplementary material for: Variations in ORAI1 Gene Associated with Kawasaki Disease
Source: PLoS One. 2016 Jan 20;11(1):e0145486. doi: 10.1371/journal.pone.0145486 (PMC4720480; doi:10.1371/journal.pone.0145486)
Supplement: S3 Fig — (PDF) [file pone.0145486.s003.pdf]

a p.G20A and p.A42_P43insPP

```
Mus musculus                12   SNPELPVSGGSSTSGSRRSRRRSGDGEPSGAPPLPPPPPAVSYPDWIGQSYSEVMSLNEH 71
Rattus norvegicus           12   SNPELPLSGGSSTSGSRRSRRRSGDGEPTGAPPLPPPP-AVSYPDWIGQSYSEVMSLNEH 70
Canis lupus familiaris      121  NSPELPLGGGSTTSGSRRSRRRSGDGEPPGSPPPP---AAVTYPDWIGQTYSEVMSLNEH 177
Felis catus                 12   NSPELPFSGGSTTSGSRRSRRRSGDGETPGSSPPP---AAVTYPDWIGQTYSEVMSLNEH 68
Bos taurus                  12   NSPELPLSGGNSTSGSRRSRRRSGDGEPPGSPPPPPP-PAVTYPDWIGQSYSEVMSLNEH 70
Sus scrofa                  12   NSPELPLSGGSTTSGSRRSRRHSGDGEPPGSPPPPPPPPAVTYPDWIGQSYSEVMSLNEH 71
Homo sapiens                12   SSPELPPSGGSTTSGSRRSRRRSGDGEPPGAPPPPP--SAVTYPDWIGQSYSEVMSLNEH 69
Gallus gallus                    ------------------------------------------------------MSLNEH 6
Xenopus laevis              7    -----------VETKSRPCSKQLQE--------------EVSYPEWISRSYVELMSLNEH 41
```

b p.S218G

```
Mus musculus                192  EVVLLCWVKFLPLKRQAGQPSPT-KPPAE-SVIVANESDSSGITPGEAAAIASTAIMVPC 249
Rattus norvegicus           191  EVVLLCWVKFLPLKRQAGQPSPT-KPPTEPAVVVANSSNNGGITPGEAAAIASTAIMVPC 249
Canis lupus familiaris      298  EVVLLCWVKFLPLKKQPGQPRPTSKPPAGSAVASVNGSSTGGITPGQAAAIASTTIMVPF 357
Felis catus                 189  EVVLLCWVKFLPLKKQPGQLRPTSKPPATDAVANVS--STGGITPGQAAAIASTTIMVPF 246
Bos taurus                  191  EVVLLCWVKFLPLKKQPGQLRPTSKPPAGGAAANVS--STGGITPGQAAAIASTTIMVPF 248
Sus scrofa                  192  EVVLLCWVKFLPLKKQPGQPRPTSKPPPSSEATNVSSSSPGGITPGQAAAIASTTIMVPF 251
Homo sapiens                190  EVVLLCWVKFLPLKKQPGQPRPTSKPPASGAAANVS---TSGITPGQAAAIASTTIMVPF 246
Gallus gallus               127  EVVLLCWVKFLPLKKNPLDPAEN---------------SNSSITSGQAAAIASTSIMVPF 171
Xenopus laevis              162  EVVLLCWVKFLPVNSPKISSNET-----------------SAVSSGQAAAITSTAIMVPF 204
```

c

	SIFT	Polyphen
p.G20A	Deleterious (0.02)	Unknown (0)
p.A42_P43insPP	NA	NA
p.S218G	Tolerated (0.5)	Benign (0.001)

S3 Fig. Prediction of impact of the amino acid sequence alterations on ORAI1 function.
(a, b) Multiple species sequence alignment of ORAI1 proteins. Positions of variants are highlighted in boldface type and yellow background color.
(c) Results of *in silico* prediction of the nature of amino acid sequence alterations. Prediction by SIFT and Polyphen-2 methods were conducted with the Variant Effect Predictor (http://ensembl.org/info/docs/tools/vep/index.html) web tool.
