# Supplementary material for: Variations in ORAI1 Gene Associated with Kawasaki Disease
Source: PLoS One. 2016 Jan 20;11(1):e0145486. doi: 10.1371/journal.pone.0145486 (PMC4720480; doi:10.1371/journal.pone.0145486)
Supplement: S4 Fig — (PDF) [file pone.0145486.s004.pdf]

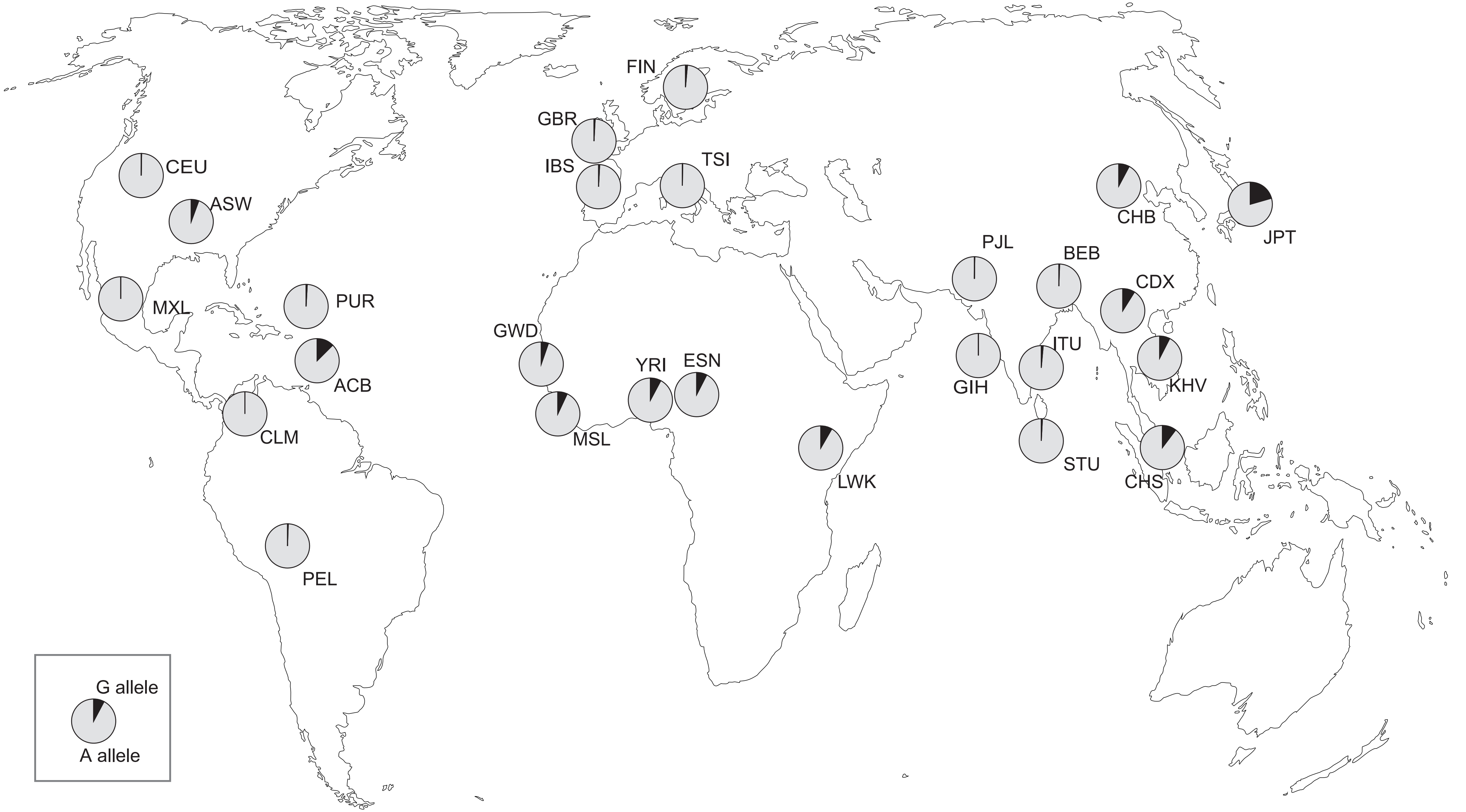

S4 Fig. Distribution of rs3741596 alleles in HapMap populations. Pie charts are demonstarting frequencies of A and G alleles in gray and black colors, respectively. CHB: Han Chinsese in Beijing, China, JPT: Japanese in Tokyo, Japan, CHS: Southern Chinese (Singapore residents of Chinese descent), CDX: Chinese Dai in Xishuang-banna, China, KHV Kinh in Ho Chi Minh City, Vietnam, CEU: Utah Residents (CEPH) with Northern and Western European Ancestry, TSI: Toscani in Italia, FIN: Finnish in Finland, GBR: British in England and Scotland, IBS: Iberian Population in Spain, YRI: Yoruba in Ibadan, Nigeria, LWK: Luhya in Webuye, Kenya, GWD: Gambian in Western Divisions in the Gambia, MSL: Mende in Sierra Leone, ESN: Esan in Nigeria, ASW: Americans of African Ancestry in SW USA, ACB: African Caribbians in Barbados, MXL: Mexican Ancestry from Los Angeles USA, PUR: Puerto Ricans from Peuto Rico, CLM: Colombians from Medellin, Columbia, PEL: Peruvians from Lima, Peru, GIH: Gujarati Indian from Houston,Texas, PJL: Punjabi from Lahore, Pakistan, BEB: Bengali from Bangladesh, STU: Sri Lankan Tamil from the UK, ITU: Indian Telugu from the UK.
